# Cardiac Tamponade Due to Purulent Pericarditis in an Immunocompetent Patient

**DOI:** 10.7759/cureus.98127

**Published:** 2025-11-30

**Authors:** Jaber El Kaissi, Najlae El Younoussi

**Affiliations:** 1 Anesthesia and Intensive Care, Moulay Ismail Military Hospital, Meknes, MAR; 2 Cardiology, Moulay Ismail Military Hospital, Meknes, MAR

**Keywords:** cardiac tamponade, drainage, pericardial effusion, point-of-care ultrasound (pocus), purulent pericarditis

## Abstract

We report the case of a previously healthy 24-year-old man admitted with a right-sided empyema and community-acquired pneumonia who subsequently developed acute chest pain, worsening dyspnea, and hemodynamic instability. Point-of-care cardiac ultrasound (POCUS) revealed a large circumferential pericardial effusion with a “swinging heart” and diastolic collapse of the right chambers, consistent with cardiac tamponade. Urgent ultrasound-guided pericardiocentesis drained thick purulent fluid, and *Streptococcus pneumoniae* was identified by polymerase chain reaction (PCR). Targeted intravenous ceftriaxone was initiated. Following drainage, the patient showed clear clinical improvement, including stabilization of blood pressure, improved oxygen saturation, reduction of tachycardia, and resolution of chest pain. Residual loculated collections required pleural drainage and a video-assisted thoracoscopic pericardial window. This case underscores the crucial role of POCUS in the early detection of cardiac tamponade in patients with empyema-associated infections and highlights the importance of rapid intervention in preventing deterioration in purulent pericarditis.

## Introduction

Purulent pericarditis is a rare but life-threatening infection representing less than 1% of pericardial diseases. Despite modern management, mortality remains high when diagnosis is delayed [1]. Before the antibiotic era, most cases resulted from direct spread of pulmonary infections, whereas today hematogenous dissemination and postoperative etiologies are more frequently implicated [1,2].

Clinical presentation is often nonspecific and may overlap with respiratory infections, which can lead to delayed recognition. Cardiac tamponade may develop rapidly and be the initial manifestation. Point-of-care ultrasound (POCUS) is a valuable bedside tool that allows early detection of pericardial effusion and hemodynamic compromise in unstable patients.

We report a case of purulent pericarditis complicated by cardiac tamponade in a previously healthy young adult with pneumonia-associated empyema. The rapid deterioration in an immunocompetent patient highlights the importance of early diagnosis and timely intervention.

## Case presentation

A previously healthy 24-year-old man presented to the emergency department with a two-day history of productive cough, right-sided pleuritic chest pain, and fever. On admission, he was alert but febrile at 39°C, with a respiratory rate of 28 breaths/min, oxygen saturation of 92% on room air, heart rate of 100 bpm, and blood pressure of 110/80 mmHg. Respiratory examination revealed markedly diminished breath sounds over the right hemithorax. Laboratory tests showed leukocytosis (24,000/mm³, 90% neutrophils) and a markedly elevated C-reactive protein (315 mg/L) (Table 1).

**Table 1:** Laboratory results on admission with corresponding reference values.

Test	Result	Reference range	Unit
White blood cell count	24,000	4000–10,000	mm³
Neutrophils	90	40–70	%
C-reactive protein (CRP)	315	<10	mg/L
Hemoglobin	13.5	13.5–17.5 (men)	g/dL
Platelets	204,000	150,000–400,000	mm³ (×10⁹/L)
Creatinine	8.05	6–12	mg/L
Urea	0.55	0.15–0.45	g/L
Sodium	142	135–145	mmol/L
Potassium	3.8	3.5–5.0	mmol/L

A chest X-ray demonstrated a large right pleural effusion with adjacent consolidation, as well as an enlarged cardiac silhouette. This enlargement was attributed either to projectional factors or to early pericardial fluid accumulation rather than intrinsic cardiomegaly, given the patient's age and clinical context. Diagnostic thoracentesis revealed frank pus consistent with empyema, and a chest tube was inserted, draining purulent fluid that later grew *Streptococcus pneumoniae*. The patient was started on intravenous amoxicillin-clavulanic acid.

Forty-eight hours after admission, the patient experienced sudden clinical deterioration characterized by central chest pain, severe dyspnea, oxygen desaturation to 76%, tachycardia (120 bpm), and hypotension (80/40 mmHg). He appeared distressed and tachypneic. Mild jugular venous distension and muffled heart sounds were noted, although pulsus paradoxus could not be reliably assessed. Because of the hemodynamic instability, an immediate point-of-care cardiac ultrasound was performed.

Echocardiography revealed a massive circumferential pericardial effusion with a classic “swinging heart” appearance and diastolic collapse of both the right atrium and right ventricle, confirming cardiac tamponade (Video 1). Internal echogenic material within the effusion suggested a complex or purulent collection, rather than a simple anechoic effusion.

**Video 1:** Circumferential pericardial effusion with “swinging heart” and diastolic collapse of the right chambers. Apical four-chamber point-of-care echocardiographic view showing a large circumferential pericardial effusion (PCE) with marked internal echogenicity, highly suggestive of a complex/purulent collection. A characteristic “swinging heart” motion is present. Right atrial (RA) and right ventricular (RV) diastolic collapse are visible, indicating tamponade physiology. LV: left ventricle. This bedside POCUS examination guided the decision for emergent pericardiocentesis. POCUS: point-of-care cardiac ultrasound.

**Figure 1:**
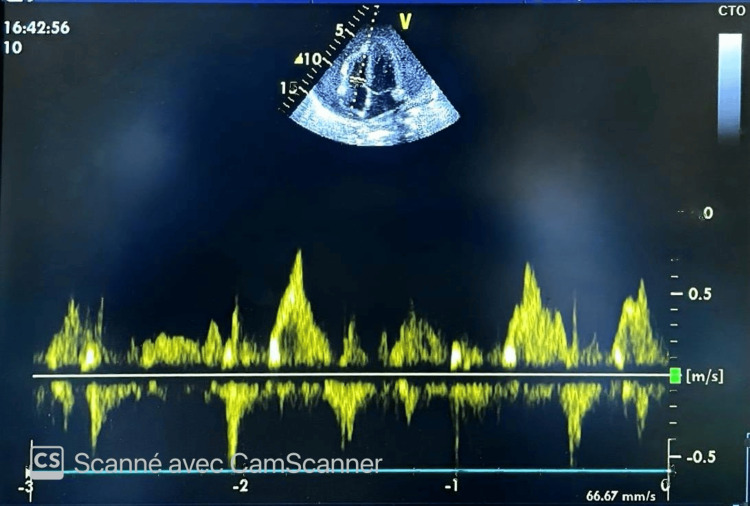
Tricuspid inflow variations. Pulsed-wave Doppler of the tricuspid inflow demonstrating marked (>50%) respiratory variation. This finding, in conjunction with right-sided chamber collapse, is highly suggestive of tamponade physiology.

**Figure 2:**
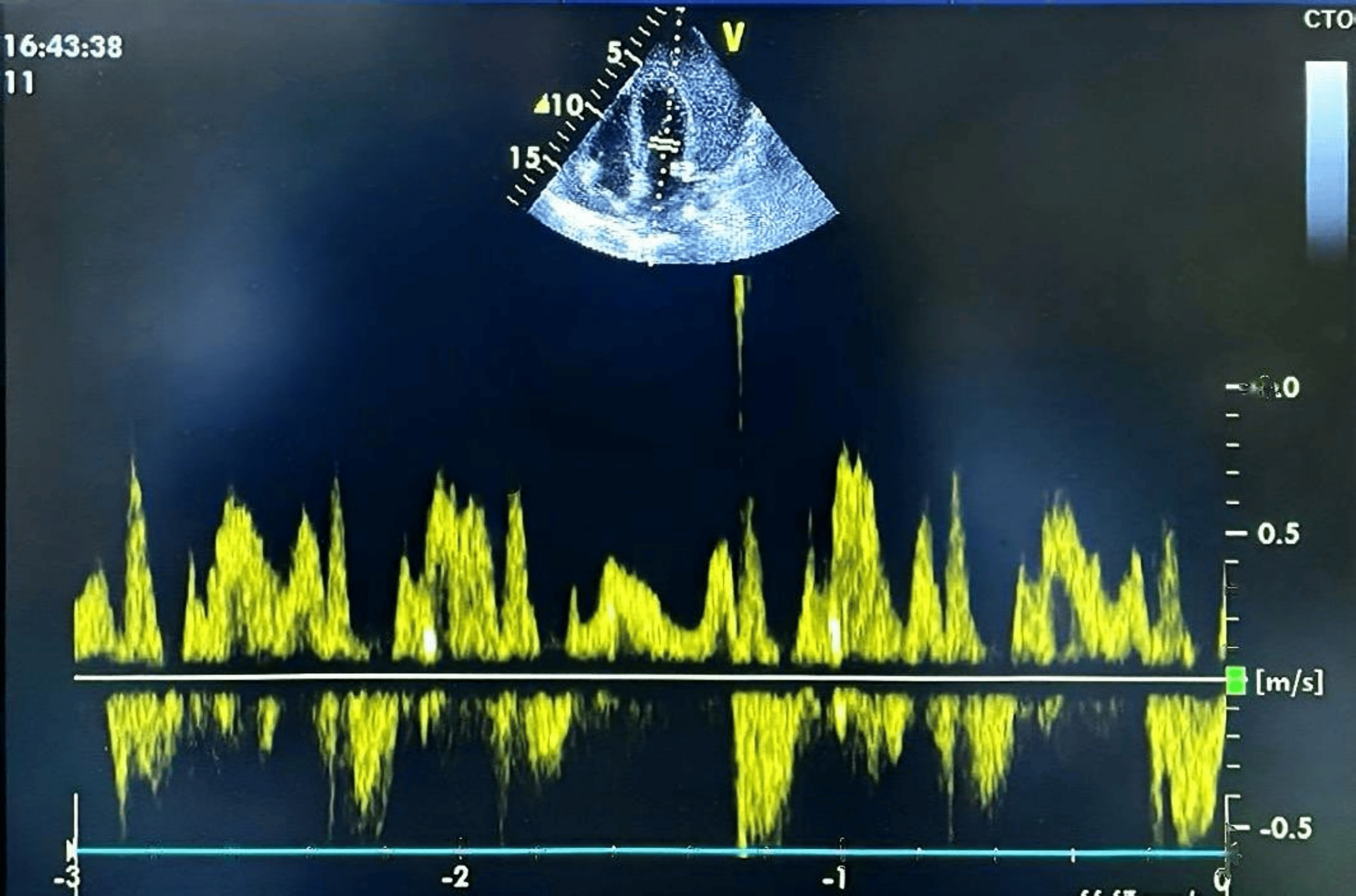
Mitral inflow variation. Pulsed-wave Doppler of the mitral inflow showing >50% respiratory variation. Combined with echocardiographic chamber collapse, these Doppler changes confirm significant respiratory dependence and support the diagnosis of cardiac tamponade.

Doppler assessment showed marked respiratory variation of more than 50% in both tricuspid and mitral inflow velocities; all Doppler measurements were obtained at the level of valvular coaptation, as recommended for accurate assessment of tamponade physiology (Figures 1, 2).

**Figure 3:**
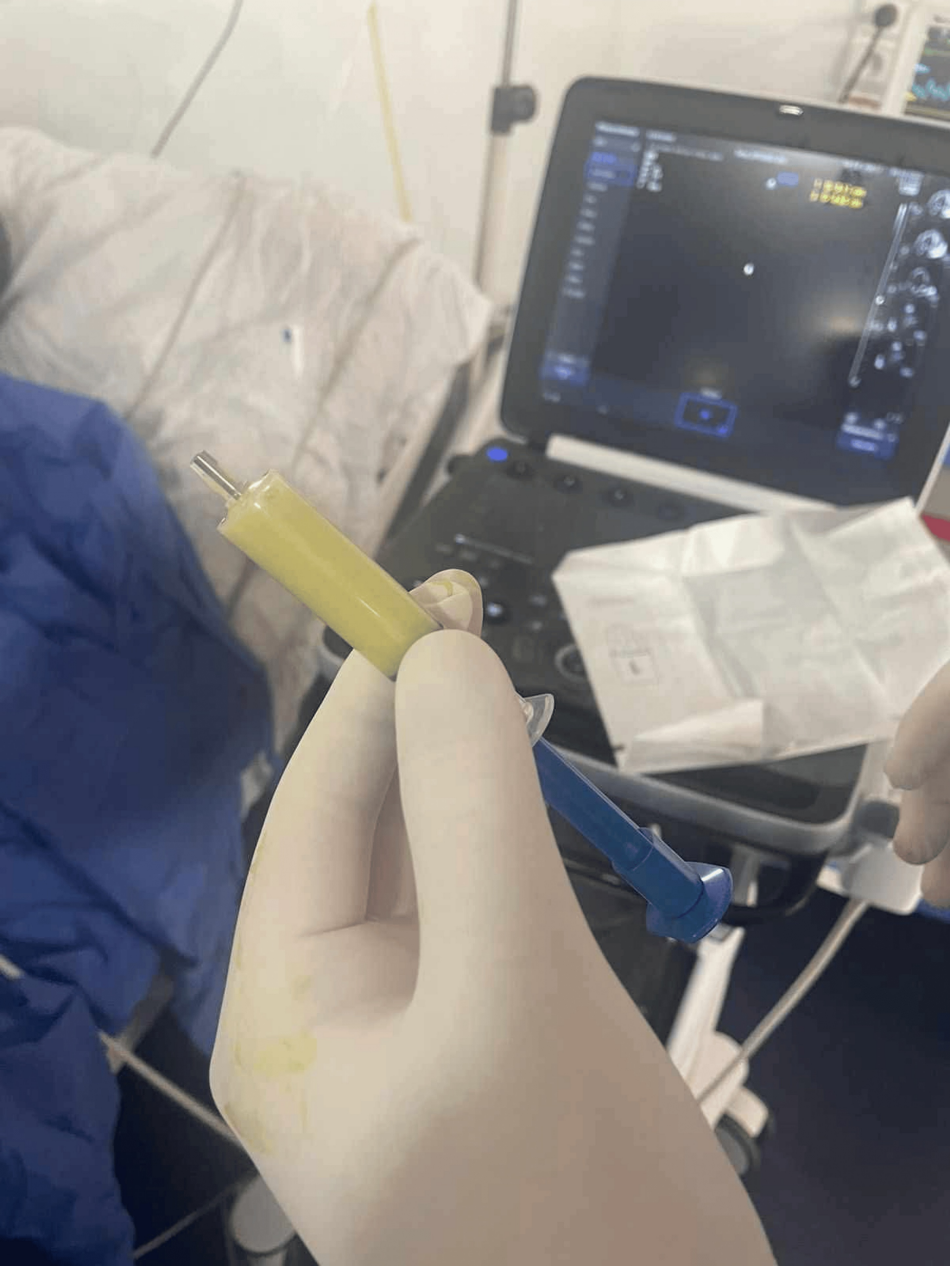
Purulent pericardial fluid. Macroscopic appearance of the pericardial fluid obtained during pericardiocentesis, showing thick, yellowish purulent material.

Urgent ultrasound-guided apical pericardiocentesis was performed using the Seldinger technique. Approximately 2.0 L of thick yellow purulent fluid was initially drained (Figure 3), and a total of 3.7 L were evacuated over the following five days. PCR testing of the pericardial fluid identified *Streptococcus pneumoniae*, matching the pleural culture. Pericardial fluid analysis revealed a markedly elevated leukocyte count with neutrophilic predominance, very low glucose levels, and significantly elevated lactate dehydrogenase (LDH), findings strongly consistent with purulent pericarditis. The patient was transitioned to intravenous ceftriaxone 2 g/day, targeted toward pneumococcal infection.

Following drainage, the patient's condition improved with progressive normalization of blood pressure, enhanced oxygenation, and resolution of chest pain. However, repeat imaging on the fourth day revealed a new multiloculated left pleural effusion as well as small residual pericardial collections, suggesting either incomplete drainage or progression of infection.

On the fifth day, the thoracic surgery team performed video-assisted thoracoscopic surgery (VATS) with drainage of the left pleural effusion and creation of a pericardial window. The patient recovered progressively and was transferred out of the intensive care unit on day seven.

## Discussion

Purulent pericarditis is an infection in the pericardial space that produces macroscopic or microscopic purulent fluid. This condition has become relatively rare with the advent of antibiotics: incidence is about 1/18,000 [1].

In a retrospective review from Spain, authors analyzed purulent pericarditis features over 20 years; they recorded only 33 cases among more than 590,000 inpatients [2].

Mechanisms have also changed with antibiotics. In the pre-antibiotic era, direct spread from an infected lung was the most common mechanism of development of purulent pericarditis. Nowadays, hematogenous spread and extension from myocardial foci or perforative injury following surgery or trauma are the most common mechanisms [3]. Also, extension from a subdiaphragmatic focus may be a rare cause [4].

The microbiologic spectrum of the disease has remained stable over the years. *Staphylococcus aureus* is the main organism involved in purulent pericarditis. However, *Streptococcus pneumoniae* is the most frequently found microorganism when there is direct spread from the lungs [5].

Clinical manifestations are variable. They are dominated by chest pain, systemic signs (fever and tachycardia), and respiratory distress. Tamponade and shock may be the primary presentation of the disease. Chest X-rays may show an enlarged cardiac silhouette, but these findings are neither specific nor sensitive.

Diagnosis of purulent pericarditis may be challenging. It is usually delayed and masked by other concomitant conditions, particularly in patients presenting with pneumonia or empyema. Because clinical manifestations can overlap, clinicians must maintain a high index of suspicion, especially when sudden hemodynamic deterioration occurs. Chest pain and general signs can also develop with pulmonary infection. Typically, a diagnosis is made when the patient shows signs of shock [6].

Echocardiography remains the cornerstone of diagnosis; it shows the pericardial effusion, assesses its size, and evaluates its hemodynamic effects. Generally, pericardial effusion appears as an anechoic image between the pericardium and epicardium in 2D cardiac ultrasound. M-mode allows a better estimation of the size of the effusion. We measure the free space between the two layers of the pericardium to classify effusion as small (less than 10 mm), medium (10-20 mm), or large (more than 20 mm). Large effusions are generally circumferential, as seen in our video.

Evaluation of hemodynamic effects allows us to search for tamponade features. The rate of accumulation of the effusion is often more important than the absolute size, and rapidly accumulating purulent effusions can cause tamponade even when the volume is moderate. The diastolic collapse of the right atrium is generally the first sign seen in this setting. It is highly sensitive but not specific to tamponade. Other signs are the diastolic collapse of the right ventricle, increased respiratory variations of mitral and tricuspid valve inflow, and dilation of the inferior vena cava [7].

Although echocardiography cannot confirm the presence of pus, internal echogenicity within the pericardial effusion is highly suggestive of a complex or purulent collection, as opposed to a simple anechoic effusion.

Purulent pericarditis should be managed aggressively, as the European Society of Cardiology recommends. Pericardial drainage and urgent antibiotics are the main treatments. Drainage should be performed without delay [8]; it allows alleviation of symptoms, ensures hemodynamic stability, and also allows pericardial pus to be obtained for bacteriological diagnosis. It is usually achieved by percutaneous pericardiocentesis, generally performed under ultrasound guidance. We can use apical or subcostal approaches or, rarely, parasternal. In our case, the effusion was circumferential and easily accessible by an apical approach. Prolonged intravenous antibiotic therapy is required, generally for a duration of three to four weeks, depending on clinical response and microbiological results.

Drainage of purulent pericarditis may not always be easy. The purulent effusion may be loculated. Some authors consider intrapericardial fibrinolysis in this setting to allow better drainage [9]. In cases when pericardiocentesis is not feasible, surgical drainage is considered.

As the follow-up showed some remaining pus with a loculated left pleural effusion, we decided to perform a pericardial window. This is a surgical procedure that involves creating a small opening in the pericardium to allow drainage and prevent reaccumulation of pus [10]. The technique was performed under VATS with the aim of draining both the left pleura and the remaining pericardial pus, allowing effective evacuation of loculated collections.

## Conclusions

Purulent pericarditis remains a rare but life-threatening complication of pneumonia, particularly when associated with empyema. This case highlights the rapid progression from pulmonary infection to cardiac tamponade in an otherwise healthy young adult, underscoring the need for a high index of suspicion when patients with pneumonia or empyema experience sudden clinical deterioration. Early repeat imaging, especially with point-of-care ultrasound (POCUS), is essential to identify evolving complications such as pericardial effusion or tamponade.

Timely pericardiocentesis, along with rapid initiation of antibiotics, can significantly improve outcomes even in severe presentations. Broader integration of POCUS into emergency and critical-care workflows, supported by adequate clinician training, may facilitate earlier recognition and intervention in similar cases.
